# Generative artificial intelligence in primary care: an online survey of UK general practitioners

**DOI:** 10.1136/bmjhci-2024-101102

**Published:** 2024-08-29

**Authors:** Charlotte R Blease, Cosima Locher, Jens Gaab, Maria Hägglund, Kenneth D Mandl

**Affiliations:** 1 Department of Women’s and Children’s Health, Uppsala University, Uppsala, Sweden; 2 Digital Psychiatry, Department of Psychiatry, Beth Israel Deaconess Medical Center, Boston, Massachusetts, USA; 3 Department of Consultation-Liaison Psychiatry and Psychosomatic Medicine, University Hospital Zurich, University of Zurich, Zurich, Switzerland; 4 Department of Clinical Psychology and Psychotherapy, University of Basel, Basel, Switzerland; 5 Computational Health Informatics Program, Boston Children’s Hospital, Boston, Massachusetts, USA

**Keywords:** Artificial intelligence, Primary Health Care, Informatics, Decision Support Techniques, Machine Learning

## Abstract

**Objectives:**

Following the launch of ChatGPT in November 2022, interest in large language model-powered chatbots has soared with increasing focus on the clinical potential of these tools. We sought to measure general practitioners’ (GPs) current use of this new generation of chatbots to assist with any aspect of clinical practice in the UK.

**Methods:**

An online survey was distributed to a non-probability sample of GPs registered with the clinician marketing service Doctors.net.uk. The study was launched as a monthly ‘omnibus survey’ which has a predetermined sample size of 1000 participants.

**Results:**

531 (53%) respondents were men, 544 (54%) were 46 years or older. 20% (205) reported using generative artificial intelligence (AI) tools in clinical practice; of those who answered affirmatively and were invited to clarify further, 29% (47) reported using these tools to generate documentation after patient appointments and 28% (45) to suggest a differential diagnosis.

**Discussion:**

Administered a year after ChatGPT was launched, this is the largest survey we know of conducted into doctors’ use of generative AI in clinical practice. Findings suggest that GPs may derive value from these tools, particularly with administrative tasks and to support clinical reasoning.

**Conclusion:**

Despite a lack of guidance about these tools and unclear work policies, GPs report using generative AI to assist with their job. The medical community will need to find ways to both educate physicians and trainees and guide patients about the safe adoption of these tools.

## Introduction

 Following the launch of ChatGPT in November 2022, interest in large language model (LLM)-powered chatbots has soared with increasing focus on the clinical potential of these tools. This new generation of chatbots are trained on vast amounts of data to generate responses, functioning like autocompletion devices. They exhibit capacities to rapidly generate and summarise text and unlike internet search engines, these models can mimic conversational interactions and ‘remember’ previous prompts.

Preliminary evidence suggests the impressive abilities of these tools to assist with writing empathic documentation,^1^ to provide more detailed documentation than typing or dictation^2^ and to assist with accurate lists of differential diagnosis.^3^ However, these tools also carry limitations. They are prone to creating erroneous information (‘hallucination’).^2^ Outputs of these models also risk perpetuating or potentially worsening racial, gender and disability inequities in healthcare (‘algorithmic discrimination’).^4^ As consumer-based applications, these tools can also risk patient privacy.^5^

Against these advances, there has been only limited measurement of the experiences and practice of clinicians. Few studies have explored doctors’ adoption of and opinions about generative artificial intelligence (AI) in clinical practice.^6^ We sought to explore general practitioners’ (GPs) use of this new generation of chatbots in the UK.

## Methods

We surveyed GPs registered with the clinician marketing service Doctors.net.uk, the largest professional network for UK doctors currently registered with the General Medical Council,^7^ with 254 741 members out of a total of approximately 379 208 registered UK doctors (67%).^8^ The study was launched as a monthly ‘omnibus survey’ which has predetermined sample sizes of 1000 participants and participants are requested to answer all closed-ended questions. The survey was pretested and piloted with 6 UK GPs (see online supplemental file 1).

All invited GPs were assured that their identities would not be disclosed to investigators and all participants gave informed consent before taking part. The survey platform www.doctors.net.uk which operates on a secure platform which ensures that personal data is numerically stored and fully anonymous (ie, not linked to the participants’ responses). All personal data such as email addresses were removed from respondents’ IDs before the transfer of the data to the research team. www.doctors.net.uk meets the requirements of the European Union Law on General Data Protection Regulation. A small incentive worth £7.50 (US$8.80, €8.83) in exchangeable shopping vouchers was provided on completion.

Sampling was stratified by regional location using demographic information about currently registered GPs working in the UK provided by the General Medical Council (GMC) in the GMC Data Explorer.^8^ Depending on GPs’ preferences for survey invitations, the study was advertised via email and/or displayed on the Doctors.net.uk homepages of selected members. During the survey administration period, approximately 21 000 GPs were active in the community. The survey ran from 2 February 2024 to 22 February 2024, closing at 1006 responses.

This study reports on Q1 of the survey (see online supplemental appendix 2); responses to other questions will be published later. In Q1, participants were asked: ‘Have you ever used any of the following to assist you in any aspect of clinical practice?’ requesting they select all that applied from: ‘ChatGPT’, ‘Bing AI’, ‘Google’s Bard’ or ‘Other’ and to specify another generative AI tool. In response to the high percentage who reported using generative AI, on 8 February 2024, after 200 responses had been gathered, we added a follow-up question for those who answered affirmatively: ‘What are you using the tools to assist with?’ Itemised survey questions with full response options and results are presented in the table 1.

**Table 1:** UK GPs’ use of generative AI in clinical practice

	Total	
	N	Percentage (%)*
‘Have you ever used any of the following to assist you in any aspect of clinical practice?’		
ChatGPT	161	16
Microsoft’s Bing AI	46	5
Google’s Bard	38	4
Other (please specify)	14	1
None	801	80
**Total**	**1006**	
‘What are you using the tools to assist with?’		
Generating documentation after patient appointments	47	29
Suggesting a differential diagnosis	45	28
Suggesting treatment options	40	25
Patient summarisation/timelines from prior documentation	32	20
Other (please specify)	53	33
Writing letters	12	(8)
**Total**	**160**	

* Since survey items requested participants select all options that applied, % does not total 100.

AI, artificial intelligence; GP, general practitioner.

## Results

Of the 1006 respondents, 531 (53%) respondents were men, 544 (54%) were 46 years or older. Describing their roles, 455 (45%) reported ‘GP Partner/Principal’, 341 (34%) ‘Salaried GP’, 181 (18%) ‘Locum GP’ and 29 (3%) ‘GP Registrar’. In online supplemental appendix 3 we describe how the respondents differ from current UK GPs. 20% (205) reported using generative AI tools in clinical practice; of those who answered affirmatively and were invited to clarify further, 29% (47) reported using these tools to generate documentation after patient appointments and 28% (45) to suggest a differential diagnosis.

## Discussion

Administered in February 2024, more than a year after ChatGPT was launched, this is the largest survey conducted into doctors’ use of generative AI in clinical practice. One in five of the UK GPs surveyed here reported using LLM-powered chatbots to assist with tasks in clinical practice; among them, ChatGPT was the most used tool. More than one in four participants who used generative AI reported using these tools to assist with writing documentation after patient appointments or with differential diagnosis.

These findings signal that GPs may derive value from these tools, particularly with administrative tasks and to support clinical reasoning. However, we caution that these tools have limitations since they can embed subtle errors and biases.^9^ They may also risk harm and undermine patient privacy since it is not clear how the internet companies behind generative AI use the information they gather. While these chatbots are increasingly the target of regulatory efforts, it remains unclear how the legislation will intersect in a practical way with these tools in clinical practice.^10^ The health industry, including electronic health record vendors, is currently grappling with how to create software that complies with safety and confidentiality standards.

The survey has limitations. Given the non-probability sample of primary care physicians who use Doctors.net.uk, this survey may not be representative of UK GPs. GPs’ decisions to respond to the topic of the survey may also have introduced biases, and it is unclear whether those more enthusiastic or those more inclined to view generative AI negatively answered the survey. Further research is needed to investigate doctors’ adoption of generative AI and how best to implement these tools safely and securely into clinical practice.

We close by noting that despite a lack of guidance about these tools and unclear work policies, GPs report using them to assist with their job. The medical community will need to find ways to both educate physicians and trainees about the potential benefits of these tools in summarising information but also the risks in terms of hallucinations, algorithmic biases and the potential to compromise patient privacy.

## Supplementary material

10.1136/bmjhci-2024-101102 online supplemental file 1

10.1136/bmjhci-2024-101102 online supplemental file 2

10.1136/bmjhci-2024-101102 online supplemental file 3
