# Supplementary material for: Generative artificial intelligence in primary care: an online survey of UK general practitioners
Source: BMJ Health Care Inform. 2024 Aug 29;31(1):e101102. doi: 10.1136/bmjhci-2024-101102 (PMC11429366; doi:10.1136/bmjhci-2024-101102)
Supplement: online supplemental file 1 [file bmjhci-31-1-s001.pdf]

Appendix 1. Checklist for Reporting Results of Internet E-Surveys (CHERRIES)

Item Category	***Checklist item***	***Described in the manuscript***	***Cited from the manuscript***
**Design**	Describe survey design	Yes	In Method section
**IRB (Institutional Review Board) approval and informed consent process**	IRB approval	Yes	Ethical approval was obtained from the Faculty of Psychology, University of Basel, Switzerland (Protocol # EKFP-034-23-1).
	Informed consent	Yes	All invited GPs were assured that their identities would not be disclosed to investigators and all participants gave informed consent before taking part.
	Data protection	Yes	All personal data such as email addresses were removed from respondents' ID before the transfer of the data to the research team. Www.doctors.net.uk meets the requirements of the EU Law on General Data Protection Regulation (GDPR).
**Development and pre-testing**	Development and testing	Yes	The survey was pretested and piloted with 6 UK GPs.
**Recruitment process and description of the sample having access to the questionnaire**	Open survey versus closed survey	Yes	We surveyed GPs registered with the clinician marketing service Doctors.net.uk, the largest professional network for UK doctors currently registered with the General Medical Council,
	Contact mode	Yes	Depending on GPs' preferences for survey invitations, the study was advertised via email and/or displayed on the Doctors.net.uk homepages of selected members.

	Advertising the survey	Yes	Depending on GPs' preferences for survey invitations, the study was advertised via email and/or displayed on the Doctors.net.uk homepages of selected members.
**Survey administration**	Web/E-mail	Both	Depending on GPs' preferences for survey invitations, the study was advertised via email and/or displayed on the Doctors.net.uk homepages of selected members.
	Context	Yes	Doctors.net.uk, the largest professional network for UK doctors currently registered with the General Medical Council,(7) with 254,741 members out of a total of approximately 379,208 registered UK doctors (67%).
	Mandatory/voluntary	Voluntary	All participants gave informed consent before taking part.
	Incentives	Yes	A small incentive worth £7.50 ($8.80, €8.83) in exchangeable shopping vouchers was provided on completion.
	Time/Date	Yes	The survey ran from February 2 to 22, 2024.
	Randomization of items or questionnaires	No	
	Adaptive questioning	Yes	In response to the high percentage who reported using generative AI, on 8 February, after 200 responses had been gathered, we added a follow-up question for those who answered affirmatively: 'What are you using the tools to assist with?'
	Number of Items	No	
	Number of screens (pages)	No	

	Completeness check	Yes	A small incentive worth £7.50 ($8.80, €8.83) in exchangeable shopping vouchers was provided on completion.
	Review step	No	
**Response rates**	Unique site visitor	No	
	View rate (Ratio of unique survey visitors/unique site visitors)	No	
	Participation rate (Ratio of unique visitors who agreed to participate/unique first survey page visitors)	No	
	Completion rate (Ratio of users who finished the survey/users who agreed to participate)	No	
**Preventing multiple entries from the same individual**	Cookies used	No	
	IP check	No	
	Log file analysis	No	
	Registration	No	
**Analysis**	Handling of incomplete questionnaires	Yes	Participants are requested to answer all closed ended questions.
	Questionnaires submitted with an atypical timestamp	No	
	Statistical correction	No	

Reference:

Eysenbach G. Improving the quality of Web surveys: the Checklist for Reporting Results of Internet E-Surveys (CHERRIES). J Med Internet Res 2004;6:e34.
