# Supplementary material for: Generative artificial intelligence in primary care: an online survey of UK general practitioners
Source: BMJ Health Care Inform. 2024 Aug 29;31(1):e101102. doi: 10.1136/bmjhci-2024-101102 (PMC11429366; doi:10.1136/bmjhci-2024-101102)
Supplement: online supplemental file 3 [file bmjhci-31-1-s003.pdf]

Appendix 3: How Respondents differ from UK GPs

Our participant pool exhibited some distinctions from the GPs registered with the GMC in the UK as of March 2024. In the GMC registry, the majority of GPs were female (40,871/72,254, 56.57%), contrasting with our sample where male respondents prevailed (531/1,006, 52.78%). Age distribution in our sample ranged from 35 and under (76/1,006, 7.5%) to 56 years or older (196/1,006, 19.48%), with the largest part aged between 36 and 45 years (386/1,006, 38.37%). Similarly, 34.59% (348/1,006) were aged 46 to 55. In comparison, the GMC registry showed a different age profile, with the majority aged between 41 and 60 years (38,775/72,254, 53.66%), while 32.13% were between 21 and 40 years old (23,218/72,254). A smaller proportion was aged 61 to 80 years (10,030/72,254, 12.88%), or 81 or above (231/72,254, 0.32%). Although proportional regional representation in our sample closely resembled that of the GMC registry, smaller variations were observed (see Table 4). Our sample exhibited slight underrepresentation in London (10,293/71,900, 14.32% vs 113/1006, 11.23%) and Scotland (6,920/71,900, 9.62% vs 84/1006, 8.35%), and slight overrepresentation in the North West (7,750/71,900, 10.78% vs 121/1006, 12.03%), North East and Yorkshire (8,683/71,900, 12.08% vs. 131/1,006, 13.02%), and the Midlands (10,328/71,900, 14.36% vs 162/1006, 16.1%).

Practice location of sample compared to GMC Registry.

	**Sample** (*n* = 1,006)	**GMC Registry** (*n* = 71,900)
**England**		
North West	121 (12.03%)	7,750 (10.78%)
North East and Yorkshire	131 (13.02%)	8,683 (12.08%)
Midlands	162 (16.1%)	10,328 (14.36%)
East of England	84 (8.35%)	6,179 (8.59%)
London	113 (11.23%)	10,293 (14.32%)
South East	130 (12.92%)	9,652 (13.42%)
South West	99 (9.84%)	6,979 (9.71%)
**Northern Ireland**	34 (3.38%)	2,075 (2.89%)
**Scotland**	84 (8.35%)	6,920 (9.62%)
**Wales**	48 (4.77%)	3,041 (4.23%)
